# The evolution of structural genomics

**DOI:** 10.1007/s12551-022-01031-8

**Published:** 2022-12-15

**Authors:** Daron M. Standley, Tokuichiro Nakanishi, Zichang Xu, Soichiro Haruna, Songling Li, Sedat Aybars Nazlica, Kazutaka Katoh

**Affiliations:** Department of Genome Informatics, Research Institute for Microbial Diseases, Osaka University, 3-1 Yamadaoka, Suita, 565-0871 Japan

**Keywords:** Structural genomics, Fold space, Protein Data Bank, Structural alignment, Protein structure prediction, Deep learning

## Abstract

Structural genomics began as a global effort in the 1990s to determine the tertiary structures of all protein families as a response to large-scale genome sequencing projects. The immediate outcome was an influx of tens of thousands of protein structures, many of which had unknown functions. At the time, the value of structural genomics was controversial. However, the structures themselves were only the most obvious output. In addition, these newly solved structures motivated the emergence of huge data science and infrastructure efforts, which, together with advances in Deep Learning, have brought about a revolution in computational molecular biology. Here, we review some of the computational research carried out at the Protein Data Bank Japan (PDBj) during the Protein 3000 project under the leadership of Haruki Nakamura, much of which continues to flourish today.

## Introduction

The term “structural genomics” appears to have been coined by Eric Lander in a 1996 Science article in which he proposed 10 goals for the post Human Genome era “With the aim of stimulating ferment” (Lander 1996). Number eight on his list was “Identification of all basic protein shapes,” which serves as a rather good working definition of structural genomics. The term itself does not appear until the conclusion of the paper, where Lander looked even further into the future: “The challenge ahead is to turn the periodic table produced by structural genomics into tools for the coming era of functional genomics.” With the number of Protein Data Bank (PDB) entries currently approaching 200,000, and the AlphaFold-EBI Protein Structure Database filling in the gaps with models for nearly every other known protein, it appears that the future that Lander was referring to in 1996 is *now*.

**Fig. 1:**
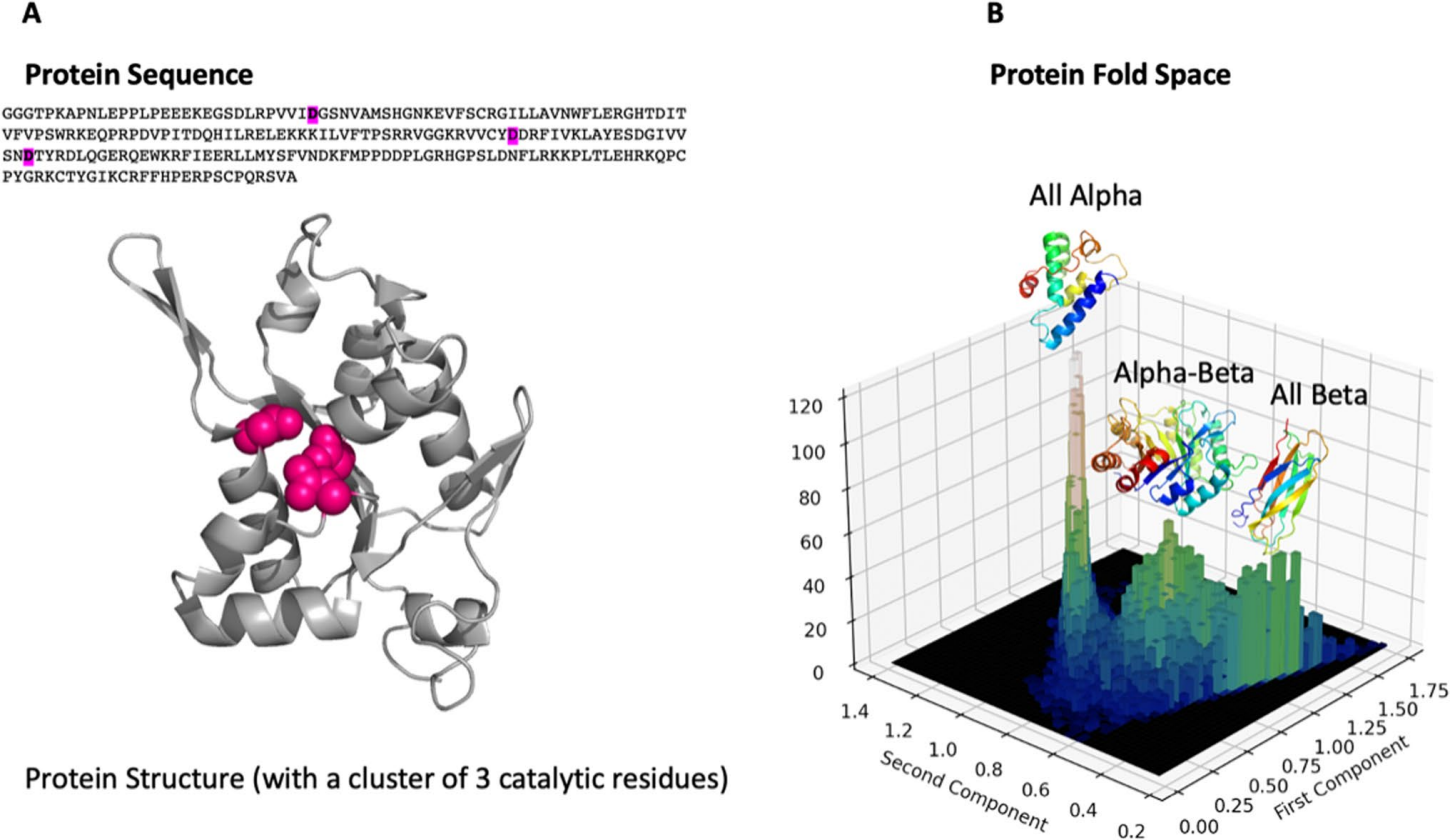
Two complementary uses of structure. **A** Structure provides a context for sequence information that allows us to envision biochemical function. Shown are the sequence and structure of the RNase domain of human Regnase-1, with catalytic aspartic acid residues highlighted. **B** Structure allows us to detect remote evolutionary relationships. Shown are all representative domains from the PDB projected into two dimensions by using a 40-dimensional feature vector comprised of a histogram of contacts between different secondary structure types. Representative structures for three of the peaks are shown. For this figure, we extracted all domains from the DASH database (described below) and described each by a feature vector consisting of the frequency of inter-residue contacts for each pair of secondary structure types defined by the DSSP program (Kabsch and Sander 1983)

As a backdrop, the Human Genome Project was described in 2011 as the “largest single undertaking in the history of biological science” (Battelle 2011). The output of this effort, which included the gene sequences coding for all known human proteins, and soon was extended to thousands of other genomes, became the input for structural genomics. Tertiary structure illuminates our understanding of biology in two complementary ways. First, it provides a frame of reference for protein sequence information derived from genomic data that can be used to understand biochemical function. That is, the structure allows us to assign a position to amino acid residues and thereby to hypothesize how a given protein works (Fig. 1A). Perturbations (e.g., by mutagenesis) allow us to check such hypotheses, and thus determine the locations of catalytic or other important residues. This structure–function paradigm has been a major driving force behind much molecular biological research.

But structure offers more than just insight into specific proteins. It also provides a means of comparing proteins that do not exhibit obvious similarity at the sequence level. This aspect of structural analysis was put very eloquently by Liisa Holm and Chris Sander: “Comparing protein shapes rather than protein sequences is like using a bigger telescope that looks farther into the universe, and thus farther back in time, opening the door to detecting the most remote and most fascinating evolutionary relations” (Holm and Sander 1996). Holm and Sander’s paper included one of the first descriptions of “fold space.” A simple rendering of the current fold space is given in Fig. 1B. Holm and Sander’s work was cited by Lander and likely strengthened the argument for structural genomics.

The logic behind structural genomics seems straightforward today, but the task of determining “all basic protein shapes” was, in the 1990s, monumental. It was also at odds with the traditional practice of protein structure determination, which emphasized the more specific, biochemical application of structure. In contrast, structural genomics focused on providing “structural coverage” of protein families with the tacit understanding that protein modeling could tackle homologous members of such families (Rost 1998). Such a selection of proteins for experimental structure determination ideally would be unbiased with regard to protein function or biological “importance.” In this regard, structural genomics represented a “major transformation” of traditional structural biology (Michalska and Joachimiak 2021).

## The PDB

It is probably safe to assume that many scientists take the existence of the PDB for granted. This is not so much a criticism of scientists as a testament to how successful the PDB has been in carrying out its mission of “maintaining a single archive of macromolecular structural data that is freely and publicly available to the global community” (Berman et al. 2003). The Worldwide PDB (wwPDB) formalized this commitment in 2003; however, the Macromolecular Structure Database (MSD) at the European Bioinformatics Institute (EBI) had been a PDB deposition center since 1998 and the Institute for Protein Research at Osaka University had been a deposition center since 2000. One of us (D.M.S.) had the privilege of working at PDB Japan (PDBj), under the direction of Haruki Nakamura, from 2003 to 2009. The PDB takes its stated mission very seriously. Both data science and experimental science have experienced rapid growth over the last few decades. Keeping up with these changes is a full-time and sometimes hectic job. The 2003–2009 period was one of the most intense because not only was PDBj establishing itself as an independent research and data center, but also structural genomics in Japan was producing a flood of new PDB entries. Under Haruki Nakamura, PDBj expanded both its deposition and research activities. On the data side, an XML schema describing PDB data was established through intensive collaboration between the three wwPDB members. To implement this new schema, a dedicated team of technicians supported the data deposition process, enriching structural data with metadata that, in turn, enriched the types of searches that could be performed as the various PDB sites.

The Protein Data Bank played several critical roles in structural genomics. Obviously, the PDB was the authoritative repository for structural data generated by the various structural genomics centers. However, a less obvious role was the importance of the PDB in protein structural modeling. By establishing a single universal repository, modelers could agree on a single ground truth for their predictions. The protein modeling community can be highly competitive. One can only imagine how difficult it would have been for participants in the Critical Assessment of protein Structure Prediction (CASP) to prepare if experimental training data was not uniform or universally accessible. Finally, because each of the wwPDB member sites developed their own structure analysis tools, PDB researchers played an important role in fairly assessing the output of structural genomics efforts.

## Structural genomics in Japan

Within the structural genomics movement, perhaps the most audacious plans were made in Japan: The stated goal of the Protein 3000 project was to solve at least 3000 new structures in 5 years. In the end, 3199 PDB entries were generated. According to an assessment by one of us (D.M.S) and Haruki Nakamura, structures for 1192 unique protein families (as given by the definition of Chandonia and Brenner (Chandonia and Brenner 2006)) were determined (Standley and Nakamura 2008). This was a significant achievement compared with the US Protein Structure Initiative, which, by the same measure, produced 597 unique families. However, the impact of these newly solved structures was not immediately felt. This is in some ways expected: many of the proteins were listed only as “domain of unknown function.” For this reason, bioinformatics tools were explicitly part of the long-term plan to reap the benefit of the newly solved structures. However, although the Protein 3000 project exceeded its numerical goals and had plans in place for future leveraging of structural information through the development of bioinformatics methods, it was the costliest Life Science project funded by the Japanese government to date, and criticism of the distribution of funds spilled into the scientific press (Cyranoski 2006; Fukushima 2016).

We are now in a position to ask whether this criticism was justified. There has been remarkable progress in protein structural modeling in the last several years (Baek et al. 2021; Jumper et al. 2021; Senior et al. 2020). The progress has been so dramatic, in fact, that many scientists have been forced to question the basic direction of their research. In this way, the AlphaFold program has been quintessentially disruptive—so much so that it is currently difficult to see the immediate future of structural biology. However, one thing is clear: the Deep Learning revolution depended directly on the existence of the structural coverage of the PDB. This coverage was the explicit goal of structural genomics. Given the benefit of this modern-day perspective, we can argue that the critics of the Protein 3000 project judged the outcome too hastily.

## Functional genomics: a case study

A protein with the obscure name of Zc3h12a illustrates how structural genomics can serve as a basis for functional genomics. Zc3h12a is a human protein of that in 2008 had an unknown structure and function. Its name was taken from the fact that it was predicted to contain a CCCH zinc finger. Zc3h12a was originally annotated as a possible transcription factor (Bidzhekov et al. 2006), but this appears to have been a false positive prediction. It was also claimed that Zc3h12a acts as a deubiquitinase (Liang et al. 2010), but this claim has not been supported. In 2008, Shizuo Akira’s lab identified Zc3h12a as a toll-like receptor-inducible gene. Moreover, Zc3h12a-knockout mice suffered from severe immune disease characterized by elevated inflammatory cytokine protein and mRNA production. Working in Haruki Nakamura’s lab, one of us (D.M.S) attempted to model the structure of a conserved region near the known zinc finger motif. This resulted in various weak hits to known structures. However, in one of these hits (PDB entry 2QIP), the identical residues were not randomly distributed; rather, they comprised an obvious cluster of aspartic acids. Although this structural hit was itself a protein of unknown function, structural alignment to other PDB entries showed that it possessed a highly similar topology to a family of ribonucleases. In this family, which included the flap endonuclease in bacterial DNA polymerase, the conserved aspartic acids coordinated Mg^2+^ and formed the site of catalytic activity. Based on this evidence, we hypothesized that zc3h12a may itself be a Mg^2+^-dependent ribonuclease, and this prediction was subsequently validated experimentally (Matsushita et al. 2009).

Now re-named “regulatory RNase 1” (Regnase-1), Zc3h12a has been found to be essential for regulating the activation of both innate and adaptive immune cells (Iwasaki et al. 2011; Uehata et al. 2013). Moreover, suppression of Regnase-1 in T cells has been shown to be an effective means of facilitating anti-tumor T-cell therapy (Wei et al. 2019). Figure 1A shows the crystal structure of the RNase domain of Regnase-1, where the catalytic aspartic acid residues can clearly be seen to form a cluster. As we can see from this example, the critical clue that suggested the correct biochemical function of Regnase-1 came through a combination of sequence-structure and structure-structure alignments. The structural alignment method used (ASH) was initially developed at PDBj for assessing structural genomics targets (Standley et al. 2004). In fact, the PDB entry that started this process, 2QIP, was a Protein 3000 target with an unknown function (VPA0982). The Regnase-1 story exemplifies the whole purpose of structural genomics: If structural biologists only studied the structures of “interesting” proteins, they would probably have skipped VPA0982, and Regnase-1 would still be known as Zc3h12a.

## The concept of structural genomics is evolving

**Fig. 2:**
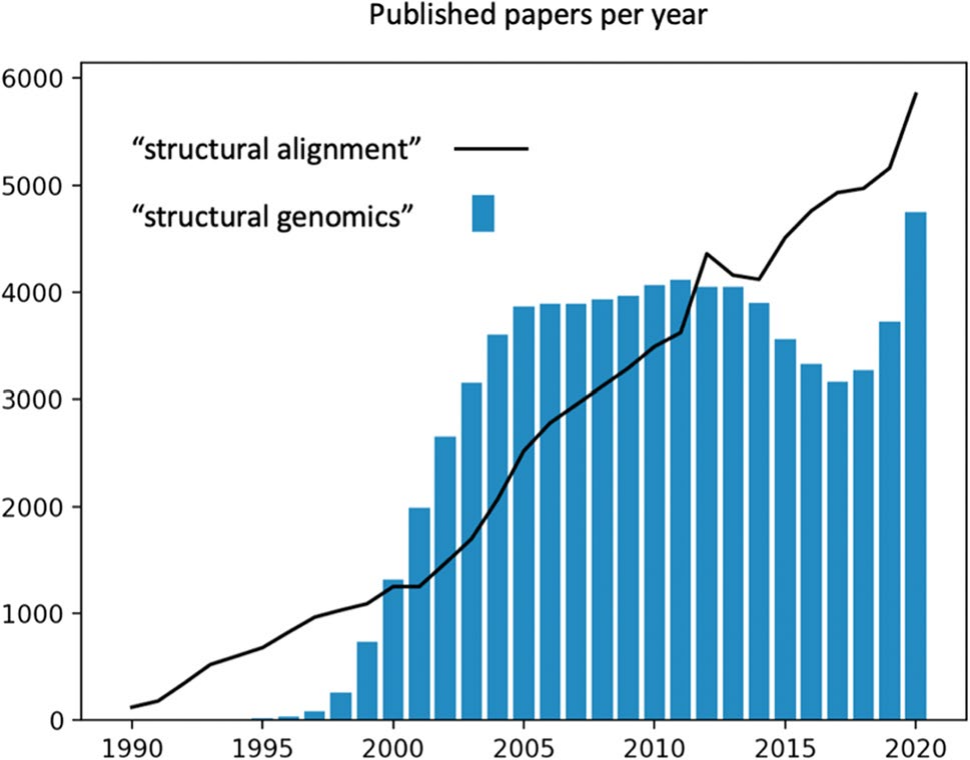
Semantic change of the term “structural genomics.” The bar graph shows the number of papers retrieved by Google Scholar using the search term “structural genomics” for each year starting with 1995 and ending with 2020. As a reference, the same search was performed using the term “structural alignment”

Interestingly, structural genomics has had an unusual trajectory in the scientific literature. In Fig. 2, we show the frequency of articles that can be retrieved on Google Scholar using the search term “structural genomics” with each calendar year, from 1990 to 2020. From 1995 to 2005, the number of such papers rose rapidly, reaching a plateau. This first broad peak makes sense because it corresponds to structural genomics funding in Japan, Europe, and the USA.

From 2013 to 2017, the number of papers dropped, but then rapidly rebounded from 2017 to the present day. As a control, we show data for the related term “structural alignment,” which exhibits a steady increase over time, as one would expect. How should the drop and then rise of structural genomics be interpreted? Based on a survey of recent literature, it appears that the meaning of structural genomics has evolved. This is a phenomenon known as “semantic change;” in the scientific literature, such change is associated with frequent use (Feltgen et al. 2017). We see that in some publications the term “structural genomics” has been repurposed to mean omics-based analysis of structure, rather than the experimental determination of structures, as a highly cited study of SARS-CoV-2 proteins exemplifies (Naqvi et al. 2020). Alternatively, the term has unexpectedly been used to refer to 3D genome architecture (Johnston et al. 2019), a completely different meaning. Interestingly, the current structural genomics wave is rising even more steeply than the original wave. It is thus interesting to speculate where structural genomics is going.

## Is there a grand unified theory of protein folding and evolution?

**Fig. 3:**
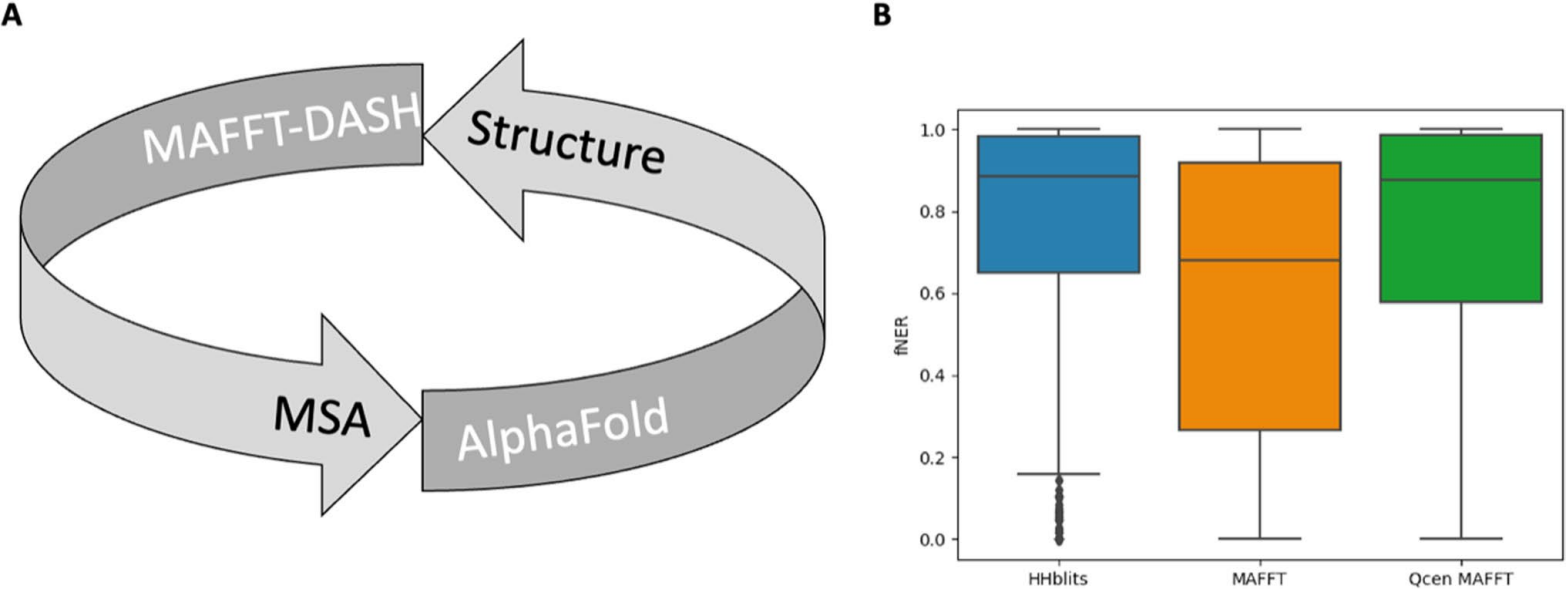
The reciprocal relationship between MSA and structure. **A** Illustration of a “closed loop” between structurally aware MSA generation and structure prediction. **B** Benchmark of the HHblits-HHpred structure prediction pipeline for 517 nonredundant (sequence identity < 60%) queries. The mean fractional number of equivalent residues (fNER) between models and the native is shown for three workflows: HHblits-HHpred (left), HHblits-MAFFT-HHpred (center), and HHblits-Qcen MAFFT-HHpred (right). In Qcen MAFFT, the target function ignores sequence pairs that do not include the query. The Wilcoxon signed-rank test *p*-values were HHBlits vs MAFFT 1.7 × 10^−42^; HHblits vs Qcen MAFFT 0.11; and MAFFT vs Qcen MAFFT 1.8 × 10^−28^). HHblits (v3.3.0), HHpred (v3.3.0), and MAFFT (v7.490) were run with default parameters. The Qcen MAFFT runs were executed after modifying the scoring function to remove non-query interactions

It seems reasonable to accept the broader meaning of structural genomics as large-scale structural analysis. We now live in an era where most structural domains have been determined experimentally or can be predicted by Deep Learning models (AlQuraishi 2021). It is interesting to speculate about what types of new challenges have been illuminated by the enhanced ability to predict protein structure. Deep Learning protein structure prediction models generally take as input a multiple alignment of sequences related to the query protein (Baek et al. 2021; Jumper et al. 2021; Senior et al. 2020). Reciprocally, multiple sequence alignment (MSA) algorithms can utilize structural information to improve accuracy (Armougom et al. 2006; Di Tommaso et al. 2011; O'Sullivan et al. 2004). As one example, we extended the ASH structural alignment package by constructing a Database of Aligned Structural Homologs (DASH), and then made DASH available to the MSA method MAFFT (Rozewicki et al. 2019). Because all the structural alignments are pre-computed, the DASH option does not add significant overhead to MAFFT and is frequently used on the MAFFT web server. Since Deep Learning models can transform MSAs into structures, and MSA algorithms can transform structures into MSAs, it is thus reasonable to conceive of a “closed loop” where predicted structures are used to improve MSAs and these MSAs are used to generate improved structures (Fig. 3A). Would such a closed loop constitute a grand unified theory of protein folding and evolution?

While this idea seems appealing, there is a small wrinkle. AlphaFold and other Deep Learning-based structure prediction methods are typically trained using MSAs that are, by evolutionary standards, poorer quality than even sequence-based MAFFT MSAs. In fact, inserting MAFFT in between HHblits (a lower-quality alignment method by evolutionary measures) and HHpred (a protein structure prediction method based on MSAs) significantly degraded the resulting model quality (Fig. 3B, left and center). We hypothesized that this phenomenon was due to the MSA target function used in MAFFT. In an evolutionarily accurate target function, all pairwise sequence relationships must be considered, and no particular sequence is more important than any other. The resulting MSAs can contain many gaps because of the difficulty in finding positions that are equivalent in all sequences. In structure prediction, however, the query sequence is clearly the most important. As a result, the MSA target function only needs to consider pairwise relationships between the query and non-query sequences. Such MSAs tend to have fewer gaps because it is easier to find equivalences between pairs of sequences than between all sequences. Indeed, if we suppress the non-query terms in the MAFFT target function (Qcen MAFFT), we find a significant improvement in HHblits-MAFFT-HHpred structural models (Fig. 3B, right). This suggests that the closed-loop concept of structure prediction and multiple alignment may work if the target function in the MSA step is adjusted to favor a single (query) sequence. Recalling Holm and Sander’s image of structural information as a more powerful telescope, we might think of query-centered multiple alignment as selecting a specific point in space for positioning this telescope.

## Antibody-antigen interactions: a special case

**Fig. 4:**
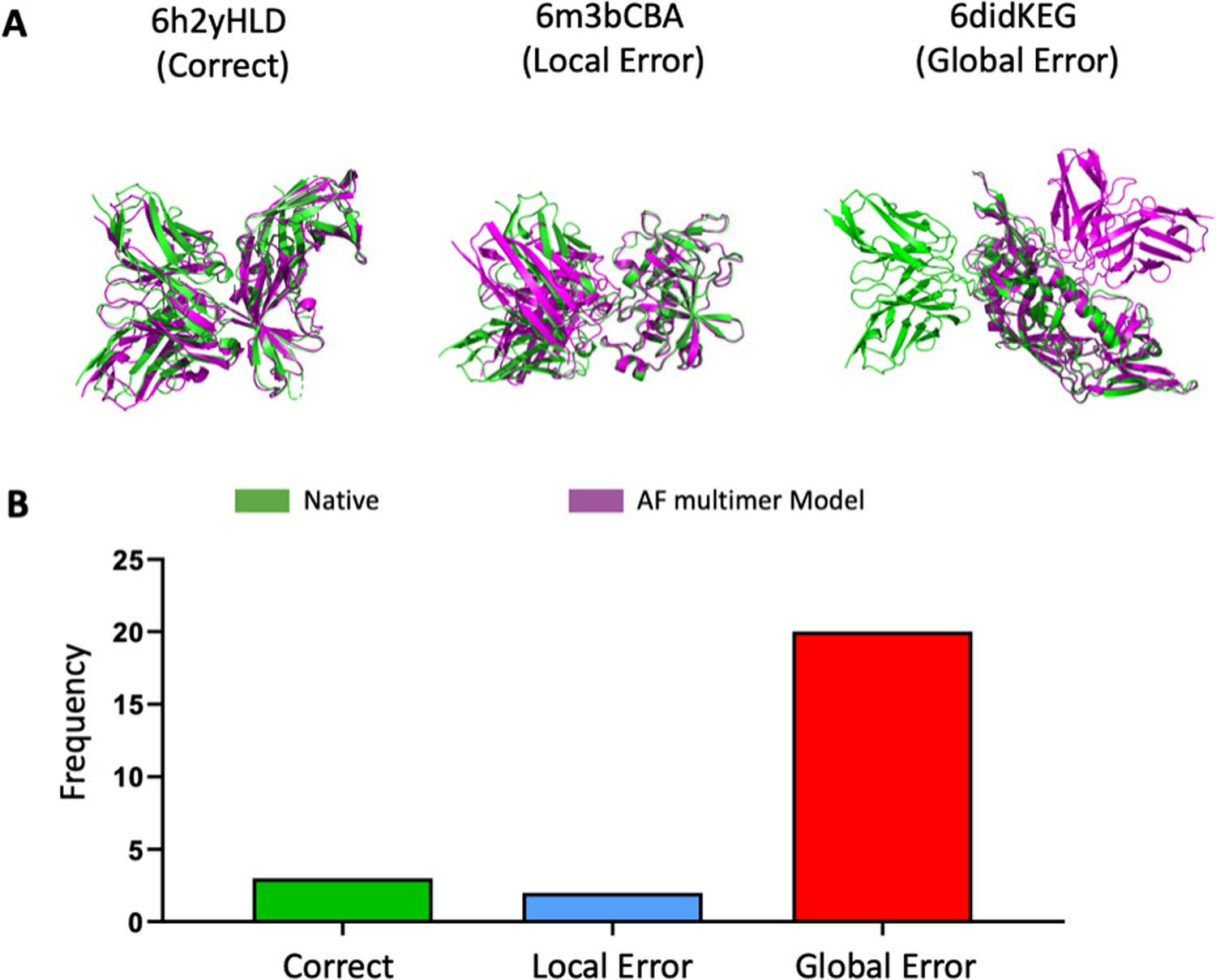
Antibody-antigen structure prediction using AlphaFold multimer. **A** Examples of correct, locally erroneous, and globally erroneous predictions are given. Here, the native is shown in green and the model in magenta. **B** Distribution of the three outcomes for a benchmark of 25 antibody-antigen complexes. Here, the AlphaFold usage and anti-RBD antibody selection were carried out as described previously (Xu et al. 2022)

Many, if not most, protein structural modeling problems fit nicely into the MSA-Deep Learning workflow described above. However, antibody-antigen interactions are an important exception. It is now well established that the extension of AlphaFold to protein complexes, AlphaFold multimer, does not work well for antibody-antigen complexes (Evans et al. 2022). Part of the problem comes from the fact that all antibodies of a given class are highly similar and can thus be aligned well in an MSA, even though, in general, they bind different antigens. Only a subset of the antibody residues—referred to as complementarity-determining regions (CDRs)—frequently interact with their cognate antigens. The combinatorial rearrangement of B cell receptor (BCR) genes, along with random mutations, give rise to a huge number (~ 10^18^) of possible antibody sequence combinations (Briney et al. 2019). This diversity is much higher than that of the sequence databases used in the training of AlphaFold multimer (Evans et al. 2022). Because different antibodies generally bind different antigens at specific binding sites (epitopes), MSAs of antibodies contain noisy information within which a coherent epitope signal is hard to detect. Indeed, when we tested AlphaFold multimer on a benchmark of 25 antibody-antigen complexes, we found that only 3 were “correct” by CAPRI (Janin et al. 2003) standards and that, of the 22 incorrect models, 20 were globally wrong in that they missed the epitope entirely (Fig. 4).

How, then, do we tackle this problem? One approach is to make use of antibody high-throughput sequencing. The number of antibody sequences deposited in public databases has grown dramatically in recent years. For example, we have recently collected over 200 million heavy chain sequences in the InterClone database (InterClone 2022). By using search and cluster functions, sequence sets with similar CDRs can easily be generated. Recent experimental work in our group has suggested that, although such CDR clusters contain antibodies that target diverse epitopes, subsets that target a common epitope of interest can be identified (Wilamowski et al. 2022). Interestingly, in a search for antibodies that target an infection-enhancing epitope on the SARS-CoV-2 spike protein, sequences acquired from COVID-19 patients were much more likely to bind the epitope than sequences acquired from healthy donors, even though the overall sequence similarities were similar in the two donor groups (Ismanto et al. 2022). We have reciprocally found that clusters of antibody or T cell receptor (TCR) sequences constitute robust feature vectors for predicting antigen exposure in donors (unpublished work). We thus propose to invest efforts in curating antibody (and TCR) sequences, including metadata on the donors’ antigen exposure, if known. This resource will hopefully play an important role in solving the antibody-antigen structure prediction problem.

### Conclusions

Large-scale science initiatives like genome sequencing and structural genomics have required huge funds and extensive coordination between scientists working in different disciplines. Such efforts also depend on prominent scientists to champion goals that may not be realized for years or decades. In the early 2000s, efforts to develop structural genomics and to expand protein structural data centers were promoted by a dedicated group of scientists with a shared vision. In Japan, Haruki Nakamura led the structural bioinformatics efforts that grew into the present-day PDBj. At the time these efforts made practical sense, but when we consider the recent transformation of computational structural biology, the development of protein sequence and structure databases seems visionary. Moreover, the combined successes of experimental and computational biology have brought us to the point where functional genomics is now possible.

In the present era, large scientific budgets are being allocated for focused research on human health. As the COVID-19 pandemic showed, we do need to overhaul our ability to rapidly roll out new vaccines and therapeutics. However, one concern is that the benefits of these large-scale projects will be assessed on a narrow timeframe, such as the immediate impact of publications. The lessons of the Protein 3000 project and PDBj are that the data itself has great value. To see the full potential of this data realized, we must give it time to evolve, just as the concept of structural genomics itself has evolved.
